# Stimulation of alpha2-adrenergic receptors impairs influenza virus infection

**DOI:** 10.1038/s41598-018-22927-0

**Published:** 2018-03-15

**Authors:** Ken Matsui, Makoto Ozawa, Maki Kiso, Makoto Yamashita, Toshihiko Maekawa, Minoru Kubota, Sumio Sugano, Yoshihiro Kawaoka

**Affiliations:** 1 Laboratory of Next Generation Drug Development, Graduate School of Frontier Sciences, University of Tokyo, Kashiwa-shi, Chiba Japan; 2 Pharmaceutical and Healthcare Research Laboratories, Research and Development Management Headquarters, Fujifilm Corporation, Kaisei-machi, Ashigarakami-gun, Kanagawa, Japan; 3 Laboratory of Animal Hygiene, Joint Faculty of Veterinary Medicine, Kagoshima University, Kagoshima-shi, Kagoshima, Japan; 4 Division of Virology, Department of Microbiology and Immunology, Institute of Medical Science, University of Tokyo, Minato-ku, Tokyo Japan; 5 Laboratory of Functional Genomics, Department of Medical Genome Sciences, Graduate School of Frontier Sciences, University of Tokyo, Minato-ku, Tokyo Japan; 6 International Research Center for Infectious Diseases, Institute of Medical Science, University of Tokyo, Minato-ku, Tokyo Japan; 7 Department of Pathobiological Sciences, School of Veterinary Medicine, University of Wisconsin-Madison, Madison, Wisconsin USA; 8 Exploratory Research for Advanced Technology Infection-Induced Host Responses Project, Japan Science and Technology Agency, Saitama, Japan

## Abstract

Influenza A viruses cause seasonal epidemics and occasional pandemics. The emergence of viruses resistant to neuraminidase (NA) inhibitors and M2 ion channel inhibitors underlines the need for alternate anti-influenza drugs with novel mechanisms of action. Here, we report the discovery of a host factor as a potential target of anti-influenza drugs. By using cell-based virus replication screening of a chemical library and several additional assays, we identified clonidine as a new anti-influenza agent *in vitro*. We found that clonidine, which is an agonist of the alpha2-adrenergic receptor (α2-AR), has an inhibitory effect on the replication of various influenza virus strains. α2-AR is a Gi-type G protein-coupled receptor that reduces intracellular cyclic AMP (cAMP) levels. In-depth analysis showed that stimulation of α2-ARs leads to impairment of influenza virus replication and that α2-AR agonists inhibit the virus assembly step, likely via a cAMP-mediated pathway. Although clonidine administration did not reduce lung virus titers or prevent body weight loss, it did suppress lung edema and improve survival in a murine lethal infection model. Clonidine may thus protect against lung damage caused by influenza virus infection. Our results identify α2-AR-mediated signaling as a key pathway to exploit in the development of anti-influenza agents.

## Introduction

Influenza viruses are a major cause of morbidity and mortality, and influenza A viruses cause annual epidemics and historically have caused pandemics with millions of deaths worldwide^1,2^. To combat influenza A viruses, two major classes of anti-influenza drugs, the neuraminidase (NA) inhibitors and the M2 ion channel inhibitors, are available in the US as Food and Drug Administration (FDA)-approved drugs for clinical use^3^. In addition, an influenza virus RNA (vRNA)-dependent RNA polymerase inhibitor is conditionally approved to treat influenza in Japan^4,5^. However, influenza A viruses mutate frequently, and both NA inhibitor- and M2 inhibitor-resistant influenza viruses have been reported^6^. The emergence of drug-resistant viruses emphasizes the need for therapeutic strategies to minimize the emergence of resistant viruses. In infectious disease, combinations of drugs with different mechanisms of action have been widely applied as a treatment approach that has produced excellent therapeutic outcomes^7,8^. Therefore, there is an urgent need to develop novel antiviral drugs that target molecules other than NA, the M2 ion channel, or the vRNA-dependent RNA polymerase to combat drug-resistant influenza viruses.

The influenza virus replication cycle is a multistep process^9^ that is initiated by virus binding to cell surface receptors. The virus then enters the cell by receptor-mediated endocytosis and is uncoated by endosomal acidification. The released viral ribonucleoprotein complexes (vRNPs) are transported into the nucleus, where vRNA transcription and replication take place. After protein synthesis and formation of new vRNP, the viral components are transported to and bud from the lipid raft microdomain on the apical plasma membrane^10,11^. Finally, progeny virions are released in an NA-dependent manner. Every step plays a vital role in the virus replication cycle, and compounds that inhibit replication steps different from those inhibited by existing drugs would likely represent useful antiviral drugs. In addition to viral proteins, numerous host factors are involved in virus replication^12–15^; the functions of most of these host factors remain largely unknown. The tyrosine/threonine kinase MEK is one of the host factors involved in virus replication, and MEK inhibitors show anti-influenza effects *in vitro* and *in vivo*^16^. Because host factors are less likely to be affected by frequent viral mutations, they are also attractive targets for the development of novel anti-influenza drugs.

There are two broad types of drug screening methods: target-based screening and phenotype-based screening. In target-based screening, the effect of compounds on a purified target protein is measured, whereas in phenotypic screening, the effect of compounds on a disease phenotype is measured. A recent statistical analysis revealed that phenotypic screening is more likely than target-based screening to contribute to the discovery of first-in-class small-molecule drugs with new molecular mechanisms of action^17^. Here, we used a high-throughput cell-based phenotypic screening approach that led us to identify a novel anti-influenza compound, clonidine. Clonidine is an α2-AR agonist and member of the Gi-type G protein-coupled receptor (GPCR) family. Stimulation of α2-ARs reduces intracellular cyclic AMP (cAMP) levels and blocks G protein signaling. In this study, we revealed that clonidine inhibits influenza virus replication by stimulating α2-ARs.

## Results

### Cell-based screening for influenza virus growth inhibitors

To detect influenza virus replication with high sensitivity, we generated a A/WSN/33 (H1N1, WSN)-based PB2-knock out influenza virus possessing the *Renilla* luciferase (Rluc) gene in the PB2 vRNA (WSN/PB2-Rluc) as described previously^18^, and constructed a cell-based screen with AX4/PB2 cells, which are MDCK cells that overexpress human 2,6-sialyltransferase and express the PB2 protein^19^. Replication of WSN/PB2-Rluc virus is restricted to AX4/PB2 cells. AX4/PB2 cells were infected with WSN/PB2-Rluc virus at a multiplicity of infection (MOI) of 0.025. To cleave the hemagglutinin (HA) of the progeny viruses and convert them to their infectious form, TPCK-treated trypsin was simultaneously added. Twenty-two hours after infection, virus replication rates were evaluated by measuring Rluc expression levels. To validate the screening assay, we confirmed the inhibitory effect of zanamivir and favipiravir (also called T-705), which are an NA inhibitor and a vRNA-dependent RNA polymerase inhibitor, respectively^20^. Zanamivir and favipiravir inhibited virus replication in a dose-dependent manner (Fig. S1); the 50% inhibitory concentrations (IC_50_s) of zanamivir and favipiravir were 3.06 nM and 2.61 µM, respectively. The IC_50_ value of zanamivir against wild-type A/WSN/33 (H1N1) was previously reported as 22 ± 10 nM^21^. The IC_50_ values of favipiravir against H1N1 wild-type viruses were also reported previously: A/PR/8/34 (1.0 µM), A/FM/1/47 (1.3 µM), A/NWS/33 (0.6 µM), A/Yamagata/120/86 (0.8 µM), and A/Suita/1/89 (0.2 µM)^4^. Our data are similar to these reported values, and thus demonstrate that virus replication inhibitors can be selected by using a cell-based screening assay with AX4/PB2 cells and WSN/PB2-Rluc virus.

**Figure 1:**
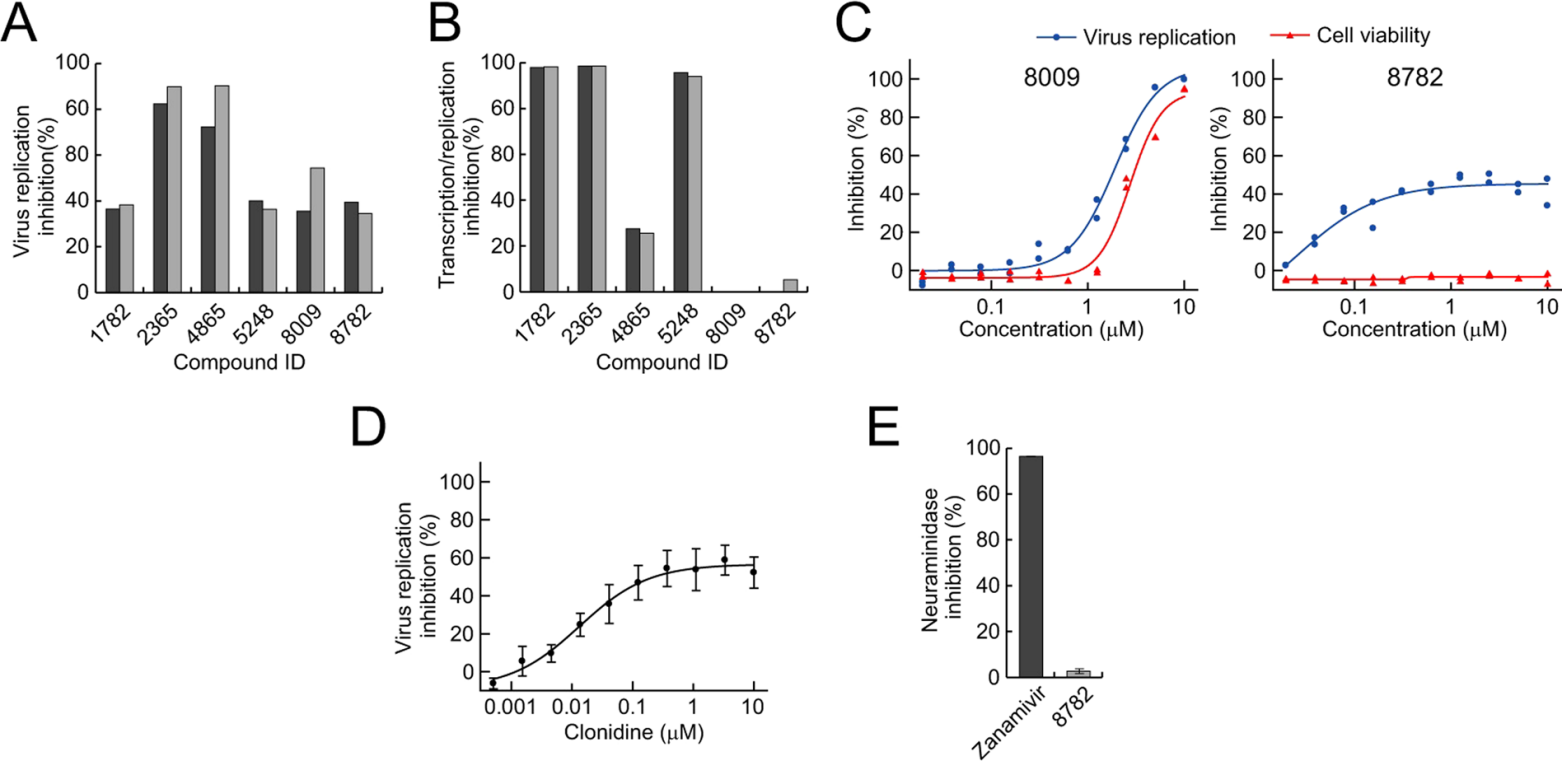
Screening for novel influenza virus replication inhibitors. (**A**) Effect of screened compounds on influenza virus replication. AX4/PB2 cells were treated with the indicated compound (1 µM each) and subjected to a virus replication assay with Rluc. Each compound was tested in duplicate assay wells. (**B**) Effect of screened compounds on influenza vRNA transcription/replication activity. 293vRNP-Puro cells were cultured with the indicated compound (10 µM each) in the presence of puromycin, and vRNA transcription/replication activity was assessed by cell viability. Each compound was tested in duplicate assay wells. (**C**) Reproducibility of virus replication inhibition and cytotoxicity of the identified compounds. AX4/PB2 cells treated with various concentrations of the indicated compounds were subjected to a virus replication assay with Rluc and a cell viability assay. Each experiment includes data from duplicate assay wells. (**D**) Effect of clonidine on influenza virus replication. AX4/PB2 cells were treated with clonidine before virus infection and subjected to a virus replication assay with Rluc. Data are shown as means ± SEM of three independent experiments. (**E**) Effect of clonidine on NA activity. WSN/PB2-Rluc virus were mixed with the indicated compounds (zanamivir, 3 nM; clonidine, 10 µM), and the NA activity of the viruses was measured with the NA-Star kit. Data are shown as means ± SEM of five independent experiments.

To select compounds that inhibit the influenza virus replication cycle, a diverse subset of 9,600 compounds from a chemical library at the University of Tokyo was screened at a final concentration of 1 µM. Six primary hit compounds (1782, 2365, 4865, 5248, 8009, and 8782) showed more than 30% inhibition in duplicate assay wells and were selected as candidates for influenza virus replication inhibitors (Figs 1A and S2). The average Z’ value was 0.80, indicating a robust assay^22^.

### Evaluation of the inhibitory effect of the candidate compounds on vRNA polymerase, cell viability, and NA

To obtain antiviral compounds with novel mechanisms of action, we first tested the inhibitory effect of the selected compounds on influenza vRNA polymerase activity by using a modified 293vRNP-Puro cell-based assay system^23^. 293vRNP-Puro cells stably express four viral proteins (i.e., PB2, PB1, PA, and NP) and a virus-like RNA encoding the puromycin resistance gene. The vRNA polymerase activity is evaluated on the basis of cell viability in the presence of puromycin. Four of the six compounds had an inhibitory effect in this vRNA transcription/replication assay (Fig. 1B), suggesting that the remaining two compounds (compound IDs, 8009 and 8782) inhibit virus replication by a mechanism different from that used by favipiravir.

In virus growth screening assays, the following three types of agents can be identified as false-positive compounds: cytotoxic agents, Rluc inhibitors, and TPCK-trypsin inhibitors. To evaluate the cytotoxic effect of compounds 8009 and 8782, we tested their inhibitory effect on influenza virus replication and AX4/PB2 cell viability at various concentrations and generated dose response curves (Fig. 1C). Compound 8782 showed dose-dependent inhibition of influenza virus replication and no cytotoxicity, whereas 8009 significantly inhibited cell viability. Therefore, we eliminated 8009 as a false-positive compound, and only 8782, clonidine (Fig. S2F), was evaluated further. To verify our screening results, the inhibitory effect of clonidine on influenza virus replication was tested with commercially available clonidine hydrochloride. The dose-response curves of 8782 (Fig. 1C) and clonidine hydrochloride (Fig. 1D) clearly overlapped, confirming that compound 8782 was clonidine. Henceforth, we used the commercial clonidine. We next tested the inhibitory effect of clonidine on Rluc activity and TPCK-trypsin activity; clonidine showed no inhibition of Rluc activity or TPCK-trypsin activity (Fig. S3). To evaluate whether four of the compounds (1782, 2365, 4865, and 5248) that showed an inhibitory effect in the vRNA transcription/replication assay (Fig. 1B) were vRNA polymerase inhibitors, additional assays were performed, specifically dose-dependent virus replication and vRNA transcription/replication assays, and cytotoxicity assays using AX4/PB2 cells and 293vRNP-Puro cells (data not shown). Compounds 1782 and 5248 showed no dose-dependent inhibition; compounds 2365 and 4865 showed cytotoxicity. These results strongly support the concept that these four compounds with inhibitory effects in the vRNA transcription/replication assay have no anti-influenza effect. Therefore, we focused on only clonidine as a true hit compound.

To examine whether clonidine inhibits influenza virus replication by inhibiting NA activity, we evaluated the NA activity of WSN/PB2-Rluc virus in the presence of zanamivir or clonidine (Fig. 1E); 3 nM zanamivir and 10 µM clonidine were equally effective at inhibiting influenza virus growth (Figs 1D and S1A). Yet, while zanamivir showed more than 90% inhibition of NA activity, clonidine had no inhibitory effect on NA activity. These results demonstrate that clonidine is an anti-influenza compound that does not inhibit NA activity or vRNA polymerase activity.

### Characterization of the inhibitory effect of clonidine on influenza virus replication

**Figure 2:**
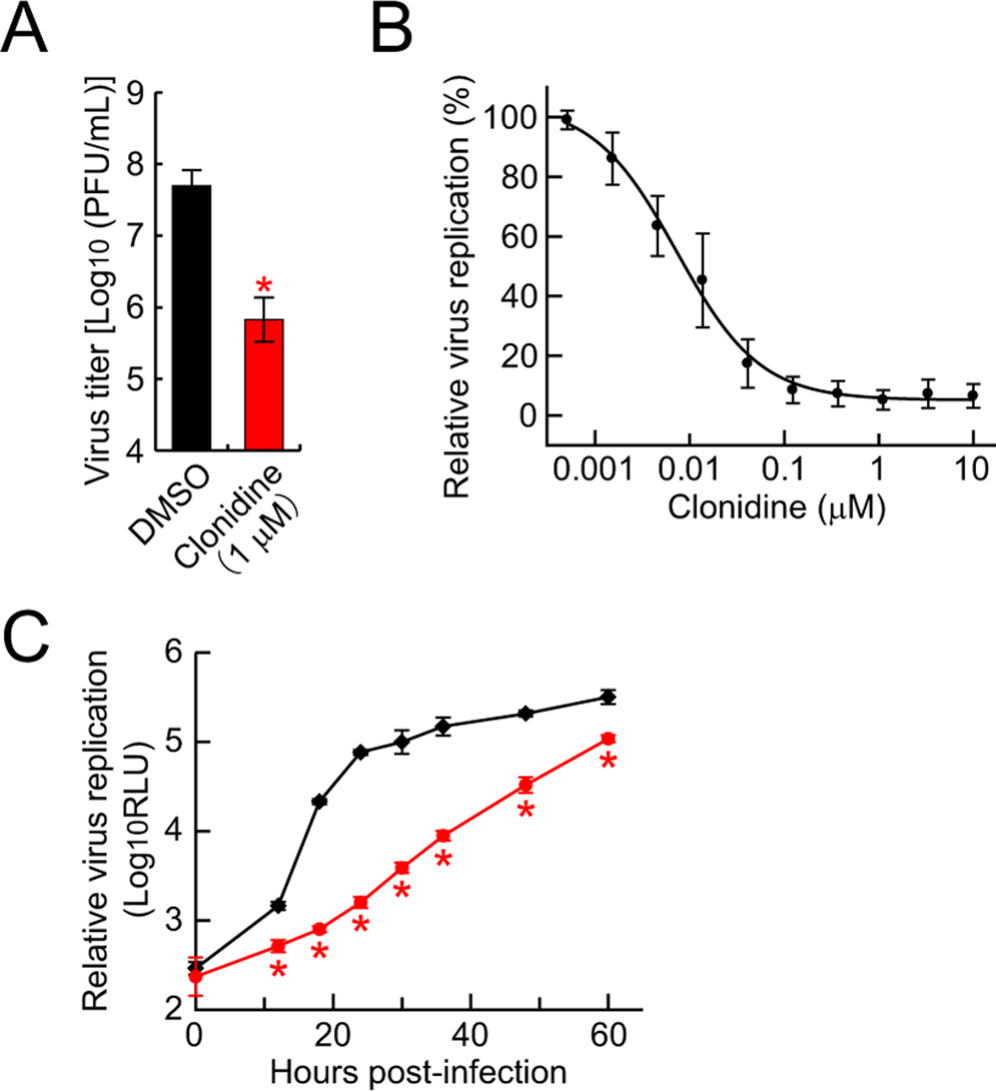
Inhibitory effect of clonidine on influenza virus replication. (**A**,**B**) Effect of clonidine on influenza virus replication. AX4/PB2 cells were treated with the indicated concentration of clonidine 1 hour before viral infection. In the presence of clonidine, the AX4/PB2 cells were infected with WSN/PB2-Rluc virus. After incubation in the presence of clonidine, virus titers were determined by using plaque assays (**A**), and NA levels in the supernatant were measured with the NA-Star kit (**B**). (**C**) Effect of clonidine on the replication kinetics of influenza viruses. AX4/PB2 cells were treated with 1 µM clonidine (Red) or DMSO (Black), and infected with WSN/PB2-Rluc virus at an MOI of 0.025. The amount of virus was determined at the indicated time points by measuring the NA levels in the supernatant. Data are shown as means ± SD of triplicate assay wells (**A** and **C**) or means ± SEM of three independent experiments (**B**). *P < 0.005.

To further validate the *in vitro* efficacy of clonidine against influenza virus replication, viruses in the culture supernatant from virus-infected cells were titrated. AX4/PB2 cells were infected with WSN/PB2-Rluc virus and incubated in the same way as in the cell-based screening assay for virus replication. Then, the virus titer in the culture supernatant was determined by using plaque assays and an NA assay; clonidine had no inhibitory effect on NA activity (Fig. 1E). Although clonidine did not completely inhibit virus replication, its presence led to an almost 2 log reduction in virus titer (Fig. 2A). Similarly by measuring NA activity in the culture supernatant as a marker of virus replication, clonidine dose-dependently decreased influenza virus replication efficiency by up to 95% (Fig. 2B). Although there is a disparity between the inhibitory effects as assessed measured by using Rluc expression levels in the host cells and the NA activity in the supernatant, this disparity can probably be attributed to the non-linear relationship between the virus-infected cell number and virus titer. To examine the effect of clonidine on virus replication kinetics, we evaluated a time-course of virus replication in both clonidine-treated cells and untreated cells by measuring the NA activity in the culture supernatant. A decrease in virus titer was observed for more than 60 h post-infection (Fig. 2C). These results, from three different assays, confirm that clonidine is an anti-influenza virus agent *in vitro*.

**Figure 3:**
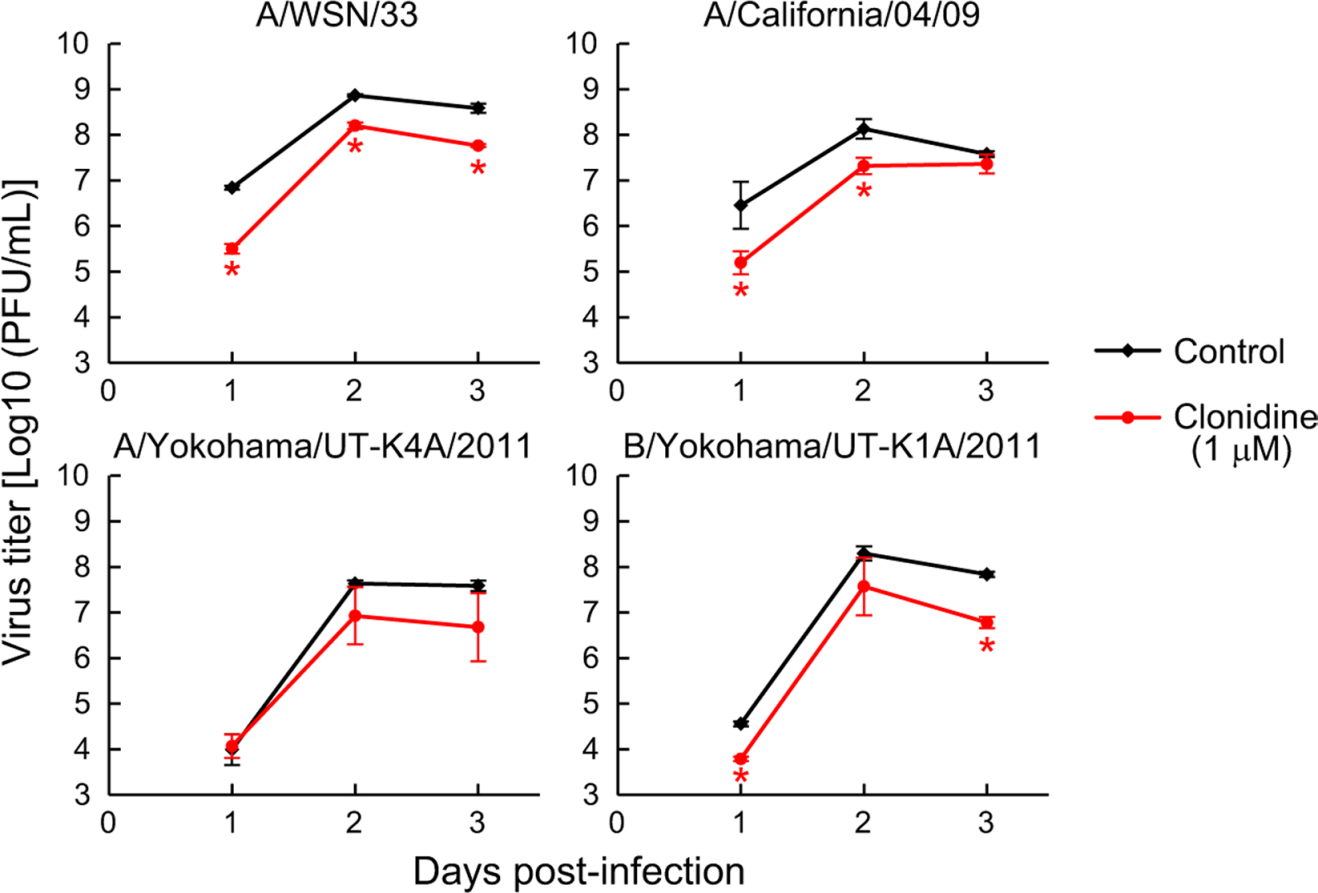
Broad-spectrum anti-influenza activity of clonidine. MDCK cells were infected with influenza A/WSN/33 (H1N1), A/California/04/2009 (H1N1pdm), A/Yokohama/UT-K4A/2011 (H3N2), and B/Yokohama/UT-K1A/2011 (Victoria lineage) at an MOI of 0.0001. The remaining viruses were removed after 1 h of the infection, and the infected cells were incubated further in the presence of clonidine. The virus titer in the supernatant was determined at the indicated time points by means of plaque assays. Data are shown as means ± SD of triplicate assay wells. *P < 0.05.

Next, to assess the spectrum of the antiviral activity of clonidine, the virus replication kinetics of the following four influenza virus strains in the presence and absence of clonidine were assessed in MDCK cells: A/WSN/33 (H1N1), A/California/04/2009 (H1N1pdm), A/Yokohama/UT-K4A/2011 (H3N2), and B/Yokohama/UT-K1A/2011 (Victoria lineage) (Fig. 3). The replication of all of virus strains tested, except for A/Yokohama/UT-K4A/2011, was significantly impaired, indicating that clonidine has broad spectrum antiviral activity against influenza viruses.

### Mechanism of action of clonidine

**Figure 4:**
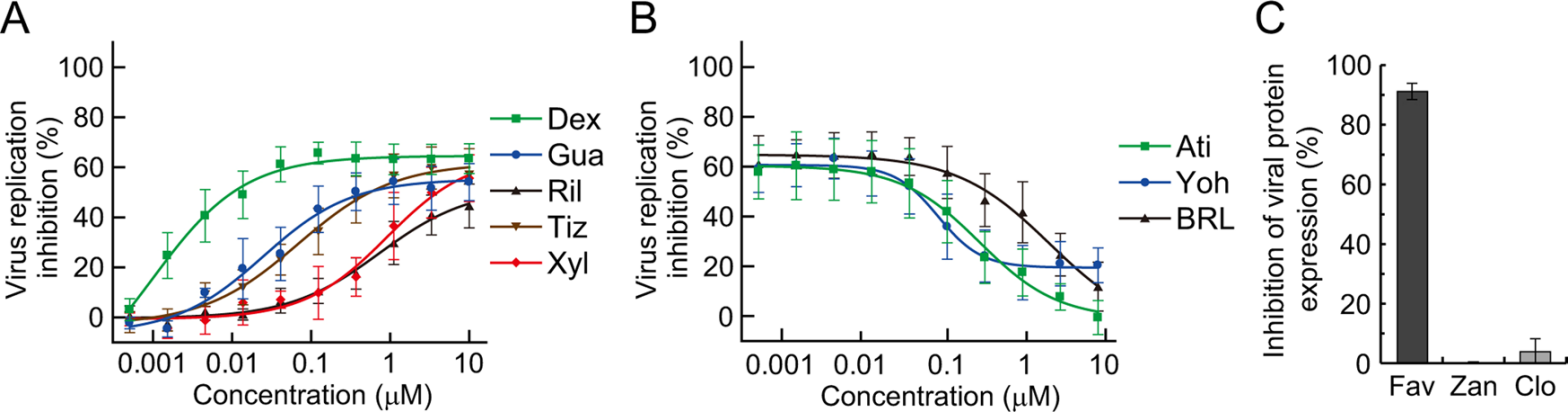
Stimulation of α2-ARs inhibits a step of the virus life cycle after protein synthesis. (**A**) Effect of α2-AR agonists on influenza virus replication. AX4/PB2 cells were treated with dexmedetomidine (Dex), guanfacine (Gua), rilmenidine (Ril), tizanidine (Tiz), or xyladine (Xyl), and subjected to a virus replication assay with Rluc. Data are shown as means ± SEM of three independent experiments. (**B**) Effect of α2-AR antagonists on clonidine-induced inhibition of virus replication. AX4/PB2 cells were treated with atipamezole (Ati), yohimbine (Yoh), or BRL-44408 (BRL) in the presence of 1 µM clonidine, and subjected to a virus replication assay with Rluc. Data are shown as means ± SEM of three independent experiments. (**C**) Effect of clonidine on viral protein expression. AX4/PB2 cells were infected with WSN/PB2-Rluc virus at an MOI of 1 and the remaining viruses were removed. After 6 h of treatment with 100 µM favipiravir (Fav), 1 µM zanamivir (Zan), or 10 µM clonidine (Clo), viral protein expression levels were assessed on the basis of Rluc expression levels. Data are shown as means ± SEM of three independent experiments.

Clonidine has been used as an antihypertensive drug, and is classified as a centrally acting α2-AR agonist^24^. To examine whether clonidine inhibits influenza virus replication through α2-ARs in host cells, we assessed the ability of several α2-AR agonists to inhibit virus replication. To minimize the possibility of inhibitory effect through molecules other than α2-ARs, we selected the following five α2-AR agonists which have diverse chemical structures: dexmedetomidine, guanfacine, rilmenidine, tizanidine, and xyladine (Fig. S4). Interestingly, virus replication was impaired in a dose-dependent manner by all five agonists (Fig. 4A), indicating the involvement of α2-ARs in the inhibition of virus replication. Furthermore, we tested the effect of α2-AR antagonists on clonidine-induced inhibition of virus replication. In the presence of 1 µM clonidine, virus replication was evaluated at various concentrations of the following three α2-AR antagonists: atipamezole, yohimbine, and BRL-44408 (Fig. 4B). The α2-AR family consists of three highly homologous subtypes: α2 A, α2B, and α2 C. Atipamezole and yohimbine are subtype-nonselective α2-AR antagonists^25^, and BRL-44408 is an α2A-selective antagonist^26^. As expected, the inhibitory effect of clonidine on virus replication was abolished by atipamezole and yohimbine. Additionally, BRL-44408 markedly reduced the inhibitory effect of clonidine on virus replication, an observation that is consistent with a previous report (note, only α2A-ARs are expressed in the MDCK-based cells)^27^. The inhibition of virus replication by dexmedetomidine, guanfacine, rilmenidine, tizanidine, and xyladine was also abolished by these α2-AR antagonists (Fig. S5). These results indicate that stimulation of a signal transduction cascade mediated by α2-ARs, especially α2A-ARs, is the basis for clonidine-induced inhibition of virus replication.

While the target molecule of clonidine was identified by using various α2-AR agonists and antagonists, the virus replication step impaired by the actions of the α2-AR agonists is unclear. Therefore, we attempted to determine the step in the virus replication cycle where inhibition occurs. We first infected AX4/PB2 cells with WSN/PB2-Rluc virus at an MOI of 1, and the remaining viruses were removed after infection. After a 6-h incubation, viral protein expression levels were measured by means of western blot analysis to examine whether α2-AR agonists inhibit the virus replication process between the adsorption and protein synthesis steps (Fig. S6). Clonidine was added before virus infection and also after removal of the virus inoculum. Since zanamivir partially inhibits the adsorption step^28^, zanamivir and favipiravir were added only after removal of the virus inoculum at concentrations that were more than 10-fold higher than the IC_50_ values determined in the virus replication assay. Among the three drugs, only favipiravir appreciably inhibited viral protein expression. To more quantitatively compare the effect of these drugs on protein expression levels, we evaluated the viral protein expression levels by using WSN/PB2-Rluc virus and measuring Rluc expression (Fig. 4C). Zanamivir did not inhibit Rluc expression, which is consistent with the known function of this drug (i.e., inhibition of virus release). By contrast, Rluc expression was strongly inhibited by favipiravir, which again is consistent with its known function (i.e., inhibition of virus polymerase activity). Under these conditions, clonidine showed no inhibitory effect on the Rluc expression level. These results suggest that α2-AR agonists inhibit steps after viral protein synthesis.

### ***In vivo*** efficacy of clonidine against influenza virus infection

**Figure 5:**
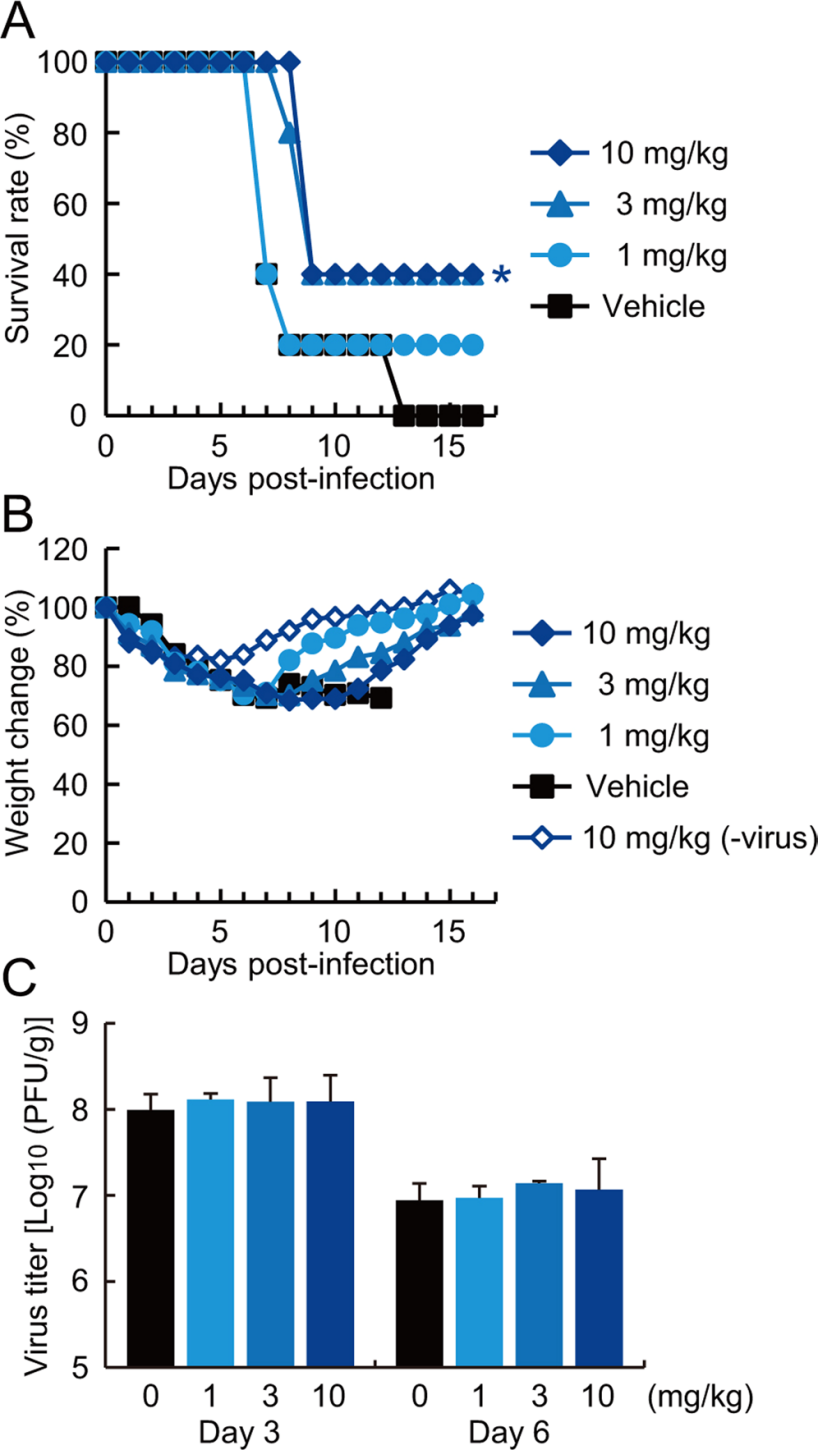
Efficacy of clonidine against H1N1 influenza viruses in mice. Five mice (**A** and **B**) and three mice (**C**) per group were intranasally infected with 370,000 pfu of mouse-adapted pandemic A/California/04/2009. The infected mice were given clonidine orally at the indicated doses once daily for 5 days beginning 2 h pi. Survival (**A**) and body weight (**B**) were monitored daily for 16 days. The survival rate was determined by death or a cut-off of 35% lost body weight. *P < 0.05. (**B**) Data are shown as means of surviving mice. (**C**) Lung virus titers on days 3 and 6 pi were determined by using plaque assays. Data are shown as means ± SD of three mice.

The α2-ARs are distributed widely throughout the body, including the brain, kidney, aorta, lung, skeletal muscle, heart, and liver^29^. Therefore, α2-AR agonists might show antiviral activity against influenza viruses *in vivo*. Among the α2-AR agonists, clonidine has an excellent pharmacokinetic profile; it is generally well-absorbed after oral administration and has a long half-life in blood^30^. To evaluate its therapeutic efficacy, we used a murine lethal infection model. Two hours post-infection (pi) with 370,000 plaque-forming units (pfu) of mouse-adapted influenza A/California/04/2009 (H1N1pdm, CA04), mice were treated with clonidine (1 mg/kg, 3 mg/kg, or 10 mg/kg) once daily for five days by oral administration. Survival and body weight of infected mice were monitored daily for 16 days, and lung virus titers were determined on days 3 and 6 pi by using plaque assays. Whereas all of the control mice died by day 13 pi, administration of 3 mg/kg or 10 mg/kg of clonidine saved 40% of the mice (Fig. 5A). By contrast, the body weights of clonidine-treated mice decreased at the same level as the vehicle control-treated, virus-infected mice (Fig. 5B). Moreover, there was no significant difference in virus titers in the lung tissues between the clonidine-treated and the untreated mice (Fig. 5C).

Although zanamivir shows poor oral bioavailability (2%), it shows excellent anti-influenza activity when administered directly to respiratory tissue^31,32^. Therefore, to distribute clonidine directly to mouse lung tissues, we next administered it intranasally. Two hours pi with 28,000 pfu of mouse-adapted CA04 influenza virus, mice were treated with clonidine (1 mg/kg, 3 mg/kg, or 10 mg/kg) once daily for five days by intranasal administration, and their survival was monitored daily for 8 days (Fig. S7A). We reduced the infectious dose used in this experiment, because we assumed that the mortality of virus-infected mice would be enhanced by intranasal administration of the drugs for the following reasons. Firstly, direct administration of drug solutions to respiratory tissue increases fluid accumulation in the lungs, leading to impairment of gas exchange^33^. Secondly, intranasally administered drug solutions deliver inoculated viruses into the deep lung, leading to spread of virus infection. Nevertheless, animals died earlier than those that received drugs orally. For this reason, lung weights and lung virus titers were measured on days 2 and 3 pi (Fig. S7B and C). Although intranasally administered clonidine provided a survival advantage, it did not reduce the virus titers in the lungs. However, there was a significant difference in lung weights between the clonidine-treated and -untreated mice (Fig. S7B), suggesting that clonidine administration suppressed pulmonary edema upon influenza virus infection. In addition, macroscopic examination revealed that the lungs of the clonidine-treated mice remained normal (data not shown). These observations suggest that although clonidine does not inhibit virus replication *in vivo*, it reduces lung edema and thereby improves the condition of the infected animals.

## Discussion

The pharmacological targeting of host factors required for virus replication is a potential therapeutic strategy for preventing the emergence of drug-resistant viruses. Genome-wide RNAi screening and other approaches have revealed that numerous host factors are involved in the influenza virus life cycle^12,13,15,16^. Here, we demonstrated that α2-AR is a host factor that plays a key role in influenza virus replication and its stimulation impairs influenza virus replication.

We found that clonidine, a well-recognized α2-AR agonist, has *in vitro* anti-influenza virus activity. Dexmedetomidine and tizanidine are also well-recognized α2-AR agonists. These three drugs inhibited virus replication at concentrations consistent with their binding affinities for α2-ARs (Figs 1D and 4A)^34^. Additionally, the rank order of their binding affinities for α2-ARs (dexmedetomidine > clonidine > tizanidine) was the same as that for their anti-influenza effects. By contrast, clonidine is also known as an I1-imidazoline receptor (I1R) agonist; the Ki for α2-AR is 32 ± 12 nM and that for I1R is 58.2 ± 17.3 nM^35,36^. In fact, most α2-AR agonists have an affinity for imidazoline receptors. Rilmenidine is also an α2-AR agonist; however, rilmenidine has a much greater affinity for I1R than for α2-AR (Ki for α2-AR, 2,440 ± 322 nM; Ki for I1R, 7.1 ± 3.5 nM)^36^. In our experiments, clonidine inhibited virus replication at an almost 100-fold lower concentration than rilmenidine (Figs 1D and 4A). This difference in inhibition concentration is similar to the difference in affinity for α2-AR but opposite to the difference in affinity for I1R. Taken together, these results indicate that α2-AR is a host factor involved in a virus replication inhibitory mechanism in MDCK-based cells.

How does α2-AR stimulation inhibit influenza virus replication? The α2-AR is a Gi-type GPCR that reduces cAMP levels through inhibition of adenylate cyclase. Exocytosis of influenza virus HA to the apical plasma membrane is retarded at low intracellular cAMP concentrations^37^. In contrast, activation of adenylate cyclase by forskolin promoted virus replication (data not shown). These findings suggest that virus replication is strongly affected by intracellular cAMP concentrations. Therefore, the delay in influenza virus replication induced by α2-AR stimulation is probably a consequence of the delay in the transport of progeny virus components containing HA that is caused by the reduction in cAMP levels. This hypothesis is consistent with our finding that clonidine did not inhibit NA activity or the processes between adsorption and viral protein synthesis (Figs 1E and 4C). Other Gi-type GPCRs may also be host factors involved in virus replication control. A previous study showed that clonidine has no effect on the decrease in cAMP in I1R-expressing cells, which do not express α2-ARs^38^. Taken together, these findings indicate that the inhibitory effect of clonidine on influenza virus replication is attributable to α2-AR stimulation.

Clonidine showed antiviral activity against a broad spectrum of influenza viruses *in vitro*, which led us to identify the responsible host factor as α2-AR. However, impairment of influenza virus replication by clonidine was not observed *in vivo*. We found that the *in vitro* inhibitory effect of clonidine on virus replication was strongly affected by medium volumes, medium change, and glucose concentrations (data not shown). The lack of effect of clonidine on virus titers in mouse lung tissues was likely due to the difference in the host cell environment. Why culture conditions affect clonidine-induced inhibition remains unclear. The extracellular glucose concentration might influence the intracellular cAMP concentration^39^. While an inhibitory effect of clonidine on influenza virus replication was not demonstrated *in vivo*, clonidine administration did significantly improve mouse survival and kept lung weights lower than those of mice given the vehicle control. Influenza virus infection causes excessive inflammatory responses including pulmonary edema, which leads to an increase in lung weights^33,40^. Although appropriate levels of cytokines in the early phase of virus infection help to control virus replication^41^, excessive amounts of cytokines lead to lung injury^42^. In addition, there is a strong negative correlation between pulmonary edema and peripheral blood oxygen saturation, and pulmonary edema and hypoxemia lead to severe influenza^40^. Therefore, clonidine administration may prolong mouse survival by protecting pulmonary function by suppressing excessive inflammatory responses or improving lung fluid clearance rather than by directly inhibiting virus replication^43^. Concomitant treatment with α2-AR agonists and anti-influenza drugs (e.g., NA inhibitors) might therefore be an effective strategy for the management of severe influenza.

In conclusion, we identified a unique host factor, α2-AR, and revealed that stimulation of α2-ARs broadly impairs influenza virus replication *in vitro*. In addition, administration of clonidine improved the survival of influenza virus-infected mice. Clonidine may be able to protect pulmonary function *in vivo*. Further investigation is required to fully understand the role of α2-ARs and other Gi-type receptors in the virus replication cycle and the protection of pulmonary function.

## Methods

### Cells and Viruses

AX4/PB2 cells were maintained in minimum essential medium (MEM; Invitrogen) supplemented with 5% (vol/vol) newborn calf serum (NCS; Invitrogen), blasticidin (10 µg/mL), and puromycin (2 µg/mL). 293vRNP-Puro cells were maintained in Dulbecco’s modified Eagle’s medium (DMEM; Lonza) supplemented with 10% fetal bovine serum (FBS; Invitrogen) and puromycin (2 µg/mL). All cells were maintained at 37 °C in 5% CO_2_. Influenza viruses A/WSN/33 (H1N1), A/California/04/2009 (H1N1pdm), mouse-adapted A/California/04/2009 (H1N1pdm), A/Yokohama/UT-K4A/2011 (H3N2), and B/Yokohama/UT-K1A/2011 (Victoria lineage) were propagated in Madin-Darby canine kidney (MDCK) cells.

### Reverse Genetics

PB2 gene-knockout (PB2KO) Renilla luciferase (Rluc)-expressing influenza virus was generated by means of plasmid-based reverse genetics^44^ as described previously^18^. Briefly, a plasmid for the expression of Rluc gene-encoding recombinant PB2 viral RNA, which contained an influenza A virus laboratory strain A/WSN/33(H1N1, WSN)-derived 3′ PB2 non-coding region, 120 nucleotides of the PB2 coding sequence at the 3′ end, the GFP coding sequence, 336 nucleotides of the PB2 coding sequence at the 5′ end, and the 5′ PB2 non-coding region, was constructed based on its GFP gene-encoding counterpart^45^. The resultant plasmid was transfected into 293 T cells with Trans-IT-293 (Mirus) together with the vRNA-expressing plasmids for the remaining seven (i.e., PB1, PA, HA, NP, NA, M and NS) genes from WSN strain and the protein-expressing plasmids for WSN-derived PB2, PB1, PA and NP proteins. The transfectant viruses were harvested at 48 h post-transfection and inoculated to AX4/PB2 cells to confirm Rluc expression in virus-infected cells and to propagate viruses. At 48–72 h after inoculation, the propagated viruses were harvested, clarified, aliquoted, and stored at −80 °C until use. The stock viruses were titrated by means of plaque assays in AX4/PB2 cells.

### Influenza Virus Replication Assay

AX4/PB2 cells in MEM containing 0.3% (wt/vol) BSA were seeded on 384-well microplates (4,000 cells/well) unless otherwise stated, and various compounds were added to the cells (10% of the total assay volume) 18 h after seeding. One hour later, WSN/PB2-Rluc virus and L-(tosylamido-2-phenyl) ethyl chloromethyl ketone (TPCK)-treated trypsin were added to the cells (20% of the total assay volume), and the assay plates were further incubated. The total assay volume was 10 µL/well, the multiplicity of infection (MOI) was 0.025, and the incubation time was 22 h, unless otherwise stated in the respective experiments. In the cell viability assay, viruses were not added to the cells. In the Rluc inhibition assay, compounds were added just before detection. Rluc activity was measured with the Renilla-Glo Luciferase Assay System (Promega). Virus contents in the cell culture supernatant were measured by means of plaque assays and with the NA-Star kit (Applied Biosystems). Cell viability was measured with the CellTiter-Glo assay system (Promega), according to the manufacturer’s instructions. Luciferase activity was measured with a microplate reader (EnVision; PerkinElmer). All dispensing processes were performed by using the Multidrop combi (Thermo) and the HORNET-NX (Wako).

For analysis of the spectrum of antiviral activity of the compound, the MDCK cell line served as the host cell. MDCK cells were seeded on 6-well microplates and were infected with various strains at an MOI of 0.0001. After a 1-h incubation, the infected cells were washed, and compound-containing medium was added to the cells. To examine virus replication kinetics, the cell culture supernatants were collected on days 1, 2, and 3 post-infection (pi). Virus titers in the supernatants were determined by use of plaque assays in MDCK cells.

### Reporter Assay for vRNA Transcription/Replication Activity

293vRNP-Puro cells were seeded with puromycin (2 µg/mL). Compounds were added to the cells and the puromycin concentration was increased to 20 µg/mL 24 h after seeding. After a 7-day incubation, cell viability was measured with the CellTiter-Glo assay system (Promega) according to the manufacturer’s instructions.

### Neuraminidase Activity Inhibition Assay

Neuraminidase activity was assessed by using the NA-Star kit (Applied Biosystems) according to the manufacturer’s instructions.

### Therapeutic Efficacy in Mice

Six-week-old female BALB/c mice (Japan SLC Inc.) were lightly anesthetized by isoflurane inhalation and intranasally inoculated with mouse-adapted influenza A/California/04/2009 (H1N1pdm) in 50 µL of PBS. Clonidine (1, 3, or 10 mg/kg) was administered orally or intranasally to groups of 5 mice 2 h pi and daily for 5 days. Mouse survival and body weight change were monitored daily. Lungs were collected and weighed and lung virus titers were determined by means of plaque assays. All mouse experiments were performed in accordance with the University of Tokyo’s Regulations for Animal Care and Use and were approved by the Animal Experiment Committee of the Institute of Medical Science, the University of Tokyo.

### Trypsin Activity Inhibition Assay

Trypsin activity was assessed by using the Proteasome-Glo Trypsin-Like Assay (Promega). Briefly, TPCK-treated trypsin and compounds were mixed in microplates, and an equal volume of Proteasome-Glo reagent, which contains 1 µM substrate, was added to the samples. Microplates were shaken, incubated at room temperature for 30 minutes, and luminescence was measured.

### Western Blot Analysis

Virus-infected AX4/PB2 cells were lysed by RIPA buffer with protease inhibitors, and the cell lysates were mixed with 4× sample buffer (Invitrogen) and 100 mM DTT. Samples were subjected to SDS/PAGE, and proteins were electrophoretically transferred to a polyvinylidene difluoride membrane (Millipore). The membrane was blocked with PVDF Blocking Reagent for Can Get Signal (Toyobo) and was then incubated with anti-influenza virus (R309) rabbit polyclonal antibodies, which were prepared in our laboratory, followed by six washes with TBS with Tween 20 (TBS-T). Finally, the membrane was incubated with a secondary antibody, AP-conjugated anti-rabbit IgG (Cell Signaling Technology), and was washed six times with TBS-T. Specific proteins were detected using 1-Step NBT/BCIP solution (Thermo). β-Tubulin as an internal control was detected with anti-β-Tubulin (Cell Signaling Technology).

### Dose Response and Statistical Analysis

Dose response curves were fitted to a sigmoidal model and IC_50_ values were calculated by using XLfit curve fitting software (IDBS). Statistically significant differences in survival rate were determined by using the log-rank test. All other data were compared by using the Student’s t-test.

## Electronic supplementary material

Supplementary Information
