# Supplementary material for: Stimulation of alpha2-adrenergic receptors impairs influenza virus infection
Source: Sci Rep. 2018 Mar 15;8:4631. doi: 10.1038/s41598-018-22927-0 (PMC5854622; doi:10.1038/s41598-018-22927-0)
Supplement: Supplementary file 1 — Supplementary Information [file 41598_2018_22927_MOESM1_ESM.docx]

Supplementary Informations for

**Stimulation of alpha2-adrenergic receptors impairs influenza virus infection**

Ken Matsui, Makoto Ozawa, Maki Kiso, Makoto Yamashita, Toshihiko Maekawa,

Minoru Kubota, Sumio Sugano, Yoshihiro Kawaoka*

*Address correspondence to Yoshihiro Kawaoka, yoshihiro.kawaoka@wisc.edu

Supplemental figure 1


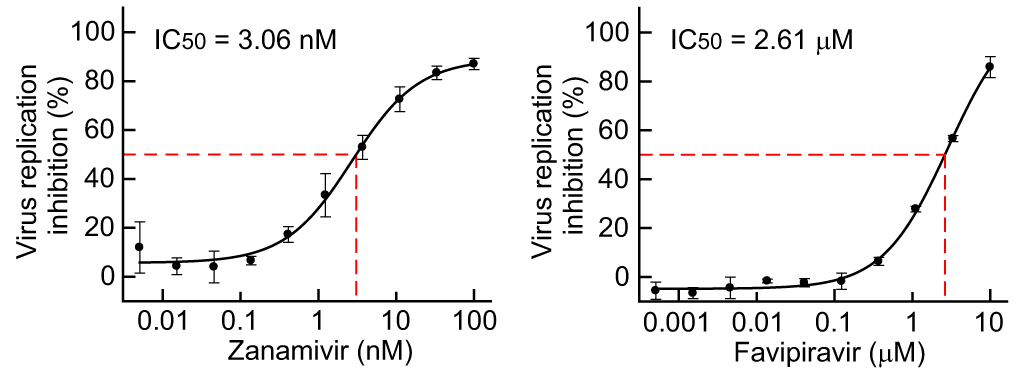


**FIG. S1.** Effect of zanamivir and favipiravir in the influenza virus replication screening system. (A and B) AX4/PB2 cells were treated with zanamivir (A) or favipiravir (B), and subjected to a virus replication assay with Rluc. Data are shown as means ± SEM of three independent experiments.

Supplemental figure 2


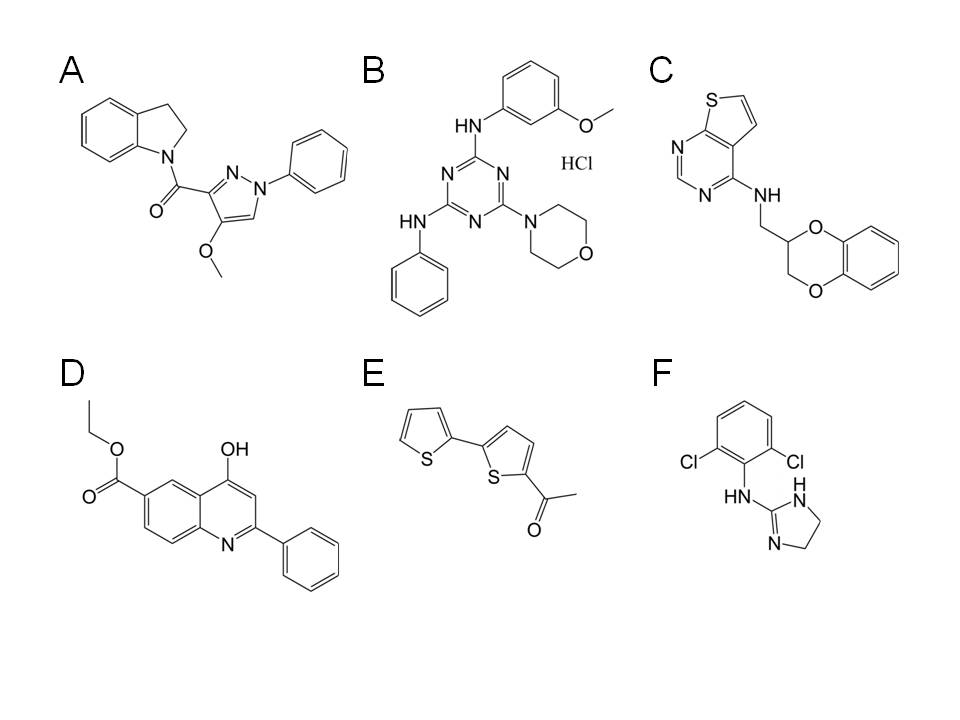


**FIG. S2.** Structures of six primary hit compounds. Compounds 1782 (A), 2365 (B), 4865 (C), 5248 (D), 8009 (E), and 8782 (F) were selected as candidate inhibitors of influenza virus replication.

Supplemental figure 3


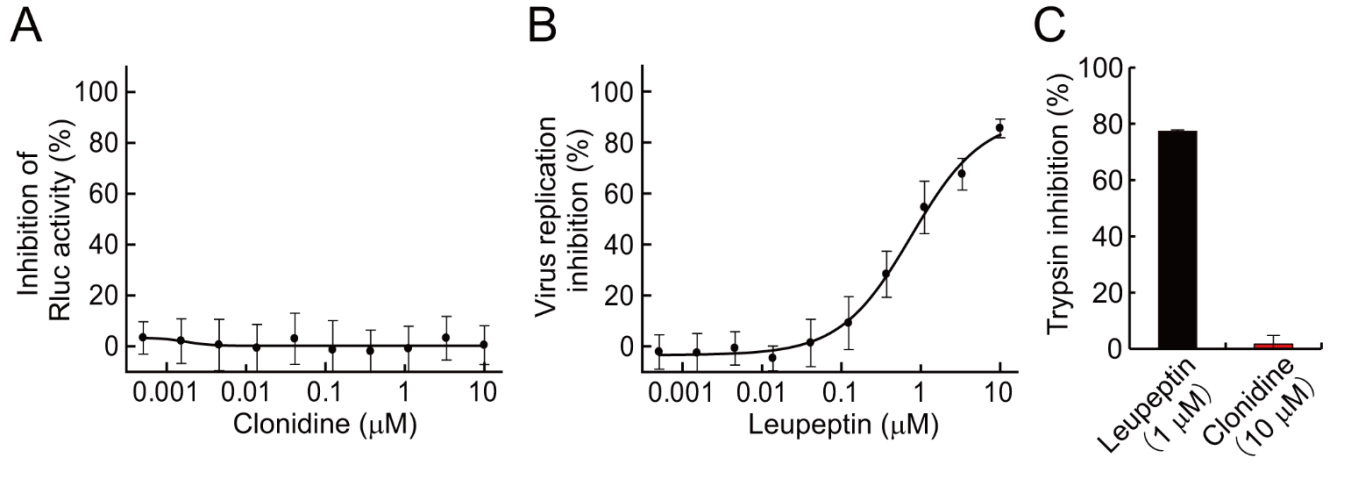


**FIG. S3.** Clonidine has no inhibitory effect on Renilla luciferase activity or trypsin activity. (A) Effect of clonidine on Rluc activity. Clonidine was added to AX4/PB2 cells infected with WSN/PB2-Rluc virus and Rluc activity was measured. Data are shown as means ± SD of sixteen assay wells. (B) Effect of leupeptin (a protease inhibitor) in the virus replication screening system. AX4/PB2 cells were treated with leupeptin, and subjected to a virus replication assay with Rluc. Data are shown as means ± SD of sixteen assay wells. (C) Effect of clonidine on trypsin activity. Leupeptin (a positive control for the inhibition of trypsin activity) or clonidine was added to TPCK-treated trypsin solution, and trypsin activity was measured with the Proteasome-Glo Trypsin-Like Assay (Promega). Data are shown as means ± SEM of three independent experiments.

Supplemental figure 4


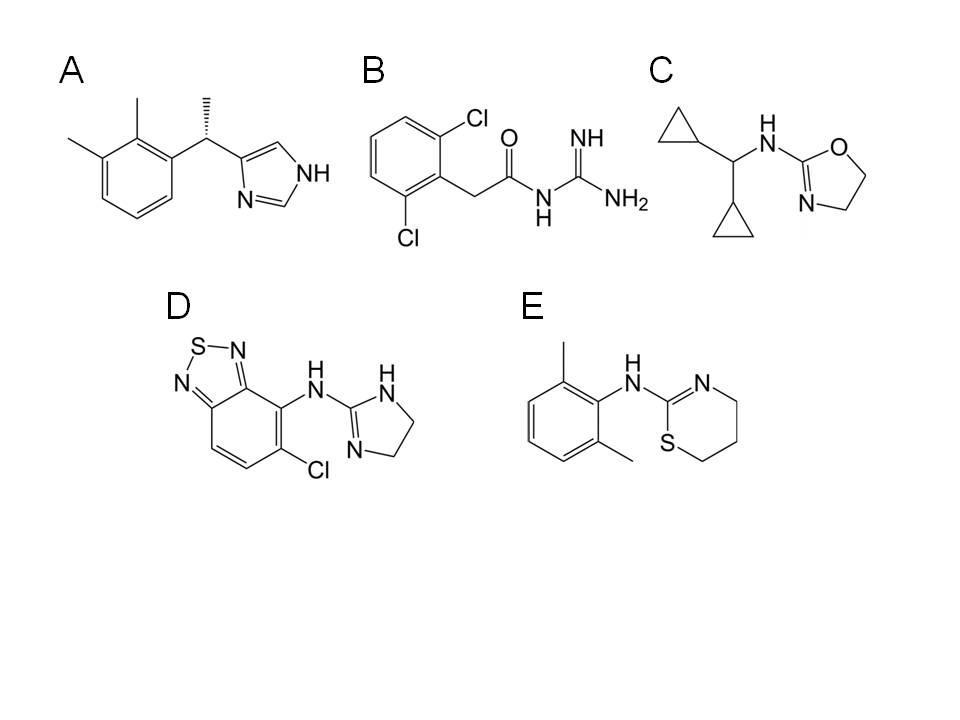


**FIG. S4.** Structures of five α2-AR agonists. Dexmedetomidine (A), guanfacine (B), rilmenidine (C), tizanidine (D), and xyladine (E) were selected as structurally diverse α2-AR agonists.

Supplemental figure 5


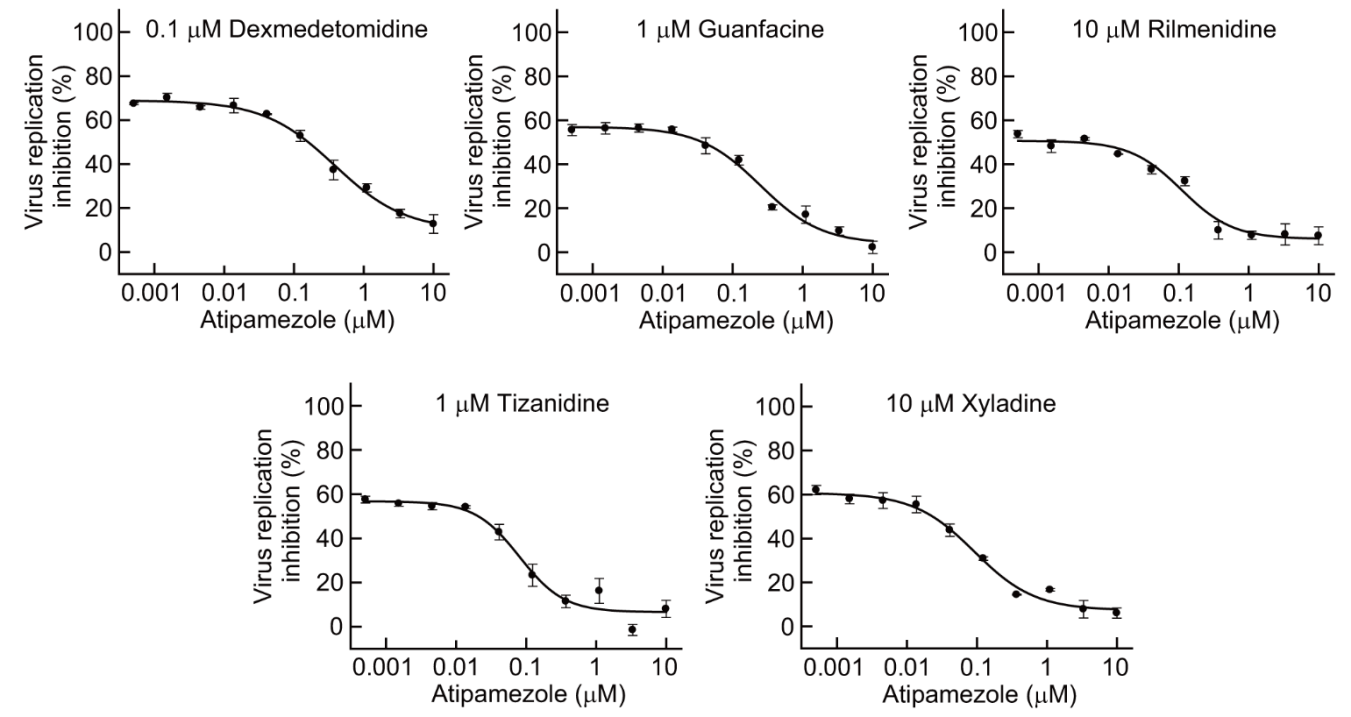


**FIG. S5.** Abolition of the virus replication inhibitory effect of α2-AR agonists by atipamezole. AX4/PB2 cells were treated with the α2-AR antagonist atipamezole in the presence of α2-AR agonists, and subjected to a virus replication assay with Rluc. Data are shown as means ± SEM of three independent experiments.

Supplemental figure 6

**FIG. S6.** Effect of clonidine on viral protein expression. AX4/PB2 cells were infected with WSN/PB2-Rluc virus at an MOI of 1 and the remaining viruses were removed. After 6 h of treatment with 100 µM favipiravir, 1 µM zanamivir, or 10 µM clonidine, viral protein expression levels were analyzed by western blotting. Viral proteins and β-Tubulin were respectively detected with anti-influenza virus polyclonal (R309) and anti-β-Tubulin by using different gels and membranes. After colorimetric detection with 1-Step NBT/BCIP solution, membranes were scanned by Satera MF8340Cdn (Canon). Middle and right panels are full-length blots. A molecular size marker (Full-Range Rainbow Molecular Weight Markers; GE Healthcare) is shown on the left in kDa.

Supplemental figure 7


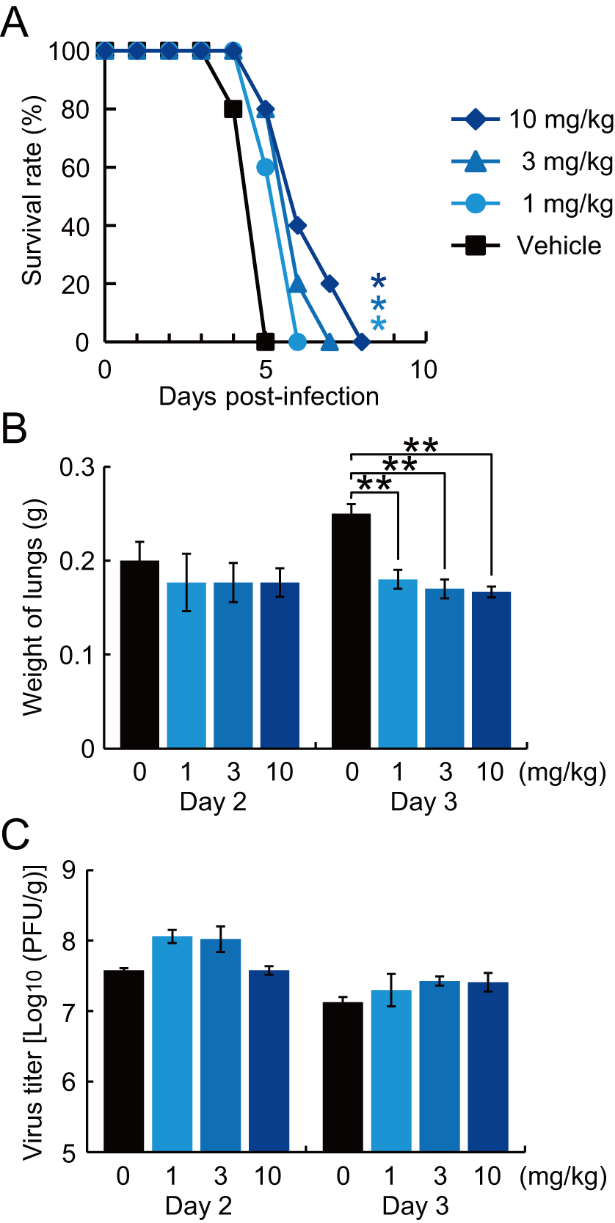


**FIG. S7.** Efficacy of intranasally administered clonidine against H1N1 influenza viruses in mice. Five mice (A) and three mice (B and C) per group were intranasally infected with 28,000 pfu of mouse-adapted pandemic A/California/04/2009. The infected mice were given clonidine intranasally at the indicated doses once daily for 5 days beginning 2 h pi. (A) Survival was monitored daily for 8 days. The survival rate was determined by death or a cut-off of 35% lost body weight. *P < 0.05. On days 2 and 3 pi, whole lungs were weighed (B) and lung virus titers were determined by using plaque assays (C). Data are shown as means ± SD of three mice. **P < 0.01.
